# Nano-topographical surface engineering for enhancing bioactivity of PEEK implants (in vitro—histomorphometric study)

**DOI:** 10.1007/s00784-023-05291-w

**Published:** 2023-10-17

**Authors:** Dawlat Mostafa, Youssef M. Kassem, Samia Soliman Omar, Yousreya Shalaby

**Affiliations:** 1 Faculty of Dentistry, Alexandria University, Alexandria, Egypt; 2 College of Dentistry, The Arab Academy for Science and Technology and Maritime Transport (AASTMT), El-Alamein, Egypt; 3 Prosthodontic Department, LSUHSC School of Dentistry, LSU Health Science Center, New Orleans, LA USA

**Keywords:** PEEK implants, Nano-topography, Nd:YAG laser, Ultraviolet irradiation, PRF

## Abstract

**Objectives:**

Dental implants are currently becoming a routine treatment decision in dentistry. Synthetic polyetheretherketone (PEEK) polymer is a prevalent component of dental implantology field. The current study aimed to assess the influence of Nd:YAG laser nano-topographical surface engineering combined with ultraviolet light or platelet rich fibrin on the bioactivity and osseointegration of PEEK implants in laboratory and animal testing model.

**Materials and methods:**

Computer Aided Design-Computer Aided Manufacturing (CAD CAM) discs of PEEK were used to fabricate PEEK discs (8 mm × 3 mm) *N* = 36 and implant cylinders (3 mm × 6 mm) *N* = 72. Specimens were exposed to Nd:YAG laser at wavelength 1064 nm, and surface roughness topography/Ra parameter was recorded in nanometer using atomic force microscopy. Laser modified specimens were divided into three groups: Nd:YAG laser engineered surfaces (control), Nd:YAG laser/UV engineered surfaces and Nd:YAG laser/PRF engineered surfaces (*N* = 12 discs–*N* = 24 implants). *In vitro* bioactivity test was performed, and precipitated apatite minerals were assessed with X-ray diffraction analysis (XRD) and scanning electron microscopy (SEM). In vivo histomorphometric analysis was performed in rabbits with BIC% calculation.

**Results:**

Ra mean value of PEEK laser engineered surfaces was 125.179 nm. For the studied groups, XRD patterns revealed distinctive peaks of different apatite minerals that were demonstrated by SEM as dispersed surface aggregations. There was a significant increase in the BIC% from control group 56.43 (0.97) to laser/UV surfaces 77.30 (0.78) to laser/PRF 84.80 (1.29) (< 0.0001).

**Conclusions:**

Successful engineered nano-topographical biomimetic PEEK implant could be achieved by Nd:YAG laser technique associated with improving bioactivity. The combination with UV or PRF could be simple and economic methods to gain more significant improvement of PEEK implant surface bioactivity with superior osteointegration.

**Supplementary Information:**

The online version contains supplementary material available at 10.1007/s00784-023-05291-w.

## Introduction

Dental implants are currently considered a routine treatment decision in dentistry. Success of dental implants is strategically based on osseointegration [1]. Currently titanium (Ti) and its alloys are the materials of choice for dental implants. However multiple issues were associated with their use [2]. Hypersensitivity to titanium is an uncommon but potential problem [3]. Another core issue is the implant stiffness which is a convenient aspect in increasing mechanical stimuli transfer to the bone when both stress and strain energy density are concerned. Correspondingly, stress shielding that could arise due to the difference in elastic moduli between metallic implants and their surrounding bone ultimately leads to peri-implant bone loss [4]. Esthetics is another disadvantage of (Ti). The dark grayish color and lack of light transmission may lead to occasional unesthetic display through thin gingival biotype [5]. Additionally, there is an increasing demand among patients for metal-free dental reconstructions [6]. Polyetheretherketone (PEEK) is another biocompatible material that was invented by DuPont (USA) [7]. PEEK is a semi crystalline polyaromatic thermoplastic polymer. Its chemical formula is (–C6H4–O–C6H4–O–C6H4–CO–)n [8]. This chemical structure helps in its superior properties including stability at high temperature (over 300 °C), resistance to chemical and radiation damage and great strength [9]. PEEK also possesses an elastic modulus of 3.6 GPa which is comparable to that of human bone [10]. PEEK is a thermoplastic high performance polymer that has been used extensively in medical treatments including spinal implants and orthopedic applications [11]. PEEK is becoming a prevalent component of dental implantology field, but its practical use suffers from several restrictions. Although PEEK is biocompatible, chemically stable, radiolucent and has an elastic modulus similar to that of normal human bone, it is biologically inert, preventing good integration with adjacent bone tissues upon implantation [12]. In order to overcome such a problem two main strategies have been suggested: either by adjusting its topographical and physicochemical properties or through combination with a bioactive coating. Surface topography of dental implants is important for adhesion and differentiation of osteoblasts during the initial phase of osseointegration as well as in long-term bone remodeling [13]. Nano-sized topographic profiles on the implant surface may play a role in the adsorption of proteins, adhesion of osteoblastic and thus increase the rate of osseointegration [14]. It was reported that nanoparticles of hydroxyapatite and calcium phosphate smaller than 100 nm could help aid creating biocompatible surfaces that aid in better osseointegration [15]. Using nano-HA has also proved to possess the ability to inhibit bacterial growth [16]. Moreover nano-HA coatings also could provide the benefit of reducing inflammatory reaction [17]. Laser treatments could be considered promising alternatives to tailor the surface topographical characteristics of PEEK to the nano-scale with subsequent enhancement of peri-implant bone healing [18]. One of the main advantages of using laser treatments is that it is accurate and precise; thus, the surface can be modified in a nano-scale without influencing the bulk properties of the material [19]. Laser surface modifications have shown to increase the wettability and biocompatibility of zirconia implants [20]. UV light has been suggested to raise the level of protein absorption and cellular attachment to implant surfaces. It was revealed that bone implant contact of implants treated with UV light was highly enhanced because of its super hydrophilicity [21], and it also reduces initial bacterial adhesions [22]. Additionally, multiple animal studies have demonstrated that UV treatment of titanium implants increased the bone-implant contact BIC% up to 98.2% [23–25]. The development of bioactive surgical additives to regulate the inflammation and increase the speed of healing process is one of the great challenges in clinical research. In this sense, platelet rich fibrin (PRF) appears as a natural and satisfactory alternative with favorable results and low risks [26]. Additionally, a study showed that the use of PRF during implant placement may enhance and increase the rate of implant osseointegration in a convenient and affordable manner [27]. Using SBF to test the in vitro bioactivity is a reliable method to evaluate their bone bonding ability, Kokubo and Takadama [28] reached the conclusion that the ability of a material to form apatite on this ability is directly related to the ability of the material to produce apatite on its surface in the living body, and bonds to living bone through this apatite layer. From these potentials, the objectives of this current study are to assess the influence of Nd:YAG laser nano-topographical surface engineering on bioactivity and osseointegration of PEEK implants and, moreover, to evaluate the impact of Nd:YAG laser combined with ultraviolet light or platelet rich fibrin on PEEK peri-implant bone integration in both laboratory and animal testing models.

## Materials and methods

### PEEK specimens fabrication

CAD CAM polymeric discs of polyetheretherketone (PEEK) (PEEK OPTIMA Juvora Ltd, Lanchire, UK) were processed according to manufacturer recommendations to fabricate PEEK discs for the in vitro study phase (8 mm × 3 mm) (*N* = 36) and implant cylinders for the in vivo study phase (3 mm × 6 mm) (*N* = 72).

### Nd:YAG laser nano-topographical engineering for PEEK specimens

The CAD CAM fabricated discs and implants of PEEK were exposed to Nd:YAG laser at wavelength 1064 nm, power 2w, 240 pulses per minute with pulse width 7 ns, repetition rate 10 Hz and the distance between laser source and disc is 30 cm for 2 min (Continuum corporate 140 Baytech Drive San Jose, CA 95134, USA). Assessment of surface roughness engineering was performed using atomic force microscopy (SPM-9700). The surface roughness topography parameter (Ra) was recorded in nanometer, and all values were automatically displayed and represented by colored 3D images for nano-topographical engineered PEEK surfaces.

### Grouping and surface modification approaches

The nano-topographical engineered PEEK specimens either discs (36) or implant cylinders (72) were divided into three equal groups: Nd:YAG laser engineered surfaces (control group), Nd:YAG laser/UV engineered surfaces group and Nd:YAG laser/PRF engineered surfaces group (*N* = 12 discs – *N* = 24 implants for each group). It is earnestly mentioned that each implant group (*N* = 24) was subdivided into two subgroups (12 implants each): one subgroup for histological assessment and the other subgroup for histomorphometric analysis (*n* = 12). The ultraviolet (UV) surface modification approach was achieved as the PEEK discs and implants with nano-topographical engineered surfaces were exposed to UV lamp at wavelength 365 nm for 48 h (Philips Lighting Company. A division of Philips Electronics North America Corporation 200 Franklin Square Drive—Somerset, NJ 08875 6800). While, the platelet rich fibrin (PRF) surface modification scheme of PEEK specimens was conducted through coating of the nano-topographical engineered PEEK surfaces with the freshly prepared PRF using the animal model of the in vivo study phase (line V Spain white rabbits) following the protocols tried to accumulate platelets and the released cytokines in a fibrin clot [26]. The animal breeding practices and animal use protocol were approved by the Institutional Animal Care and Use Committee (ALEXU-IACUC) 111907143.

### *In vitro* study phase (biomimetic–bioactivity test)

All groups of nano-topographical engineered PEEK discs were subjected to the biomimetic–bioactivity test including: Nd:YAG laser engineered surfaces (control group), Nd:YAG laser/UV engineered surfaces and Nd:YAG laser/PRF engineered surfaces (*N* = 12 discs). Simulated body fluid (SBF) was freshly prepared according to Kokubo protocol [28] to act as a biomimetic environment into which each specimen was soaked at 37 °C for 4 weeks to assess its bioactive properties. Subsequently, in vitro bioactivity assessment was accomplished to investigate apatite minerals precipitated on the surfaces of studied discs. X-ray diffraction analysis (XRD) (PANalytical (Holand), X Pert PRO) was conducted to predict the phase crystallography of calcium phosphate minerals precipitants; furthermore, scanning electron microscopy (SEM) (JEOL JSM-5300- JSM, Tokyo, Japan) was operated at 25 kV after gold sputter-coating to inspect surface morphology.

### *In vivo* study phase design in rabbit model

Thirty-six male line V Spain white rabbits were obtained from the Poultry Research Center, Faculty of Agriculture, Alexandria University (six months old and 3 kg) in good health and randomly divided into 3 groups (*N* = 12) comparable to the implant groups as follows: Nd:YAG laser engineered surfaces (control group), Nd:YAG laser/UV engineered surfaces group and Nd:YAG laser/PRF engineered surfaces group. Two implants were inserted in the femurs of each rabbit: one inserted into the distal head of right femur and the other into the left head. This animal study phase was conducted according to the guidelines approved by the Institutional Animal Care and Use Committee (ALEXU-IACUC) 111907143.

### Surgical protocol for implant placement

All surgical procedures were performed under general anesthesia and aseptic conditions. Rabbits were anesthetized with intramuscular injection of ketamine with xylazine at a dose of 35 mg/kg and 5 mg/kg of body weight, respectively. Reflection of a surgical flap was performed to expose the femur distal head, then sequential drilling of implant socket was performed under sufficient cooling at room temperature with an absolute minimum amount of trauma. Each implant was inserted followed by repositioning and suturing of the surgical flap. It is praiseworthy mentioned that during surgical insertion of the Nd:YAG laser/PRF engineered implants, PRF was freshly prepared and each implant was completely immersed into the PRF gel followed by its insertion with full packing of PRF gel into the implant socket. Postoperative intramuscular injection of broad-spectrum antibiotic and analgesic was administrated every 72 h for 10 days. Rabbits were monitored daily for weight gain and cage behavior. Implants were allowed to heal for 6 weeks before sacrifice [29].

### Histological assessment and histomorphometric analysis

At the end of the experimental periods, all the animals were sacrificed, and then as previously mentioned, each animal group either the Nd:YAG laser engineered control group and the other two laser/UV or laser/PRF groups was subsequently subdivided into two subgroups reflecting the results of (12) implants for each subgroup as follows (*n* = 12); the decalcified histological sections of the first subgroup were prepared for histological examination, while those of the second subgroups were prepared as undecalcified sections for histomorphometric analysis through calculation of the bone-to-implant contact percentage (BIC%).

### Histological examination

Following animal sacrification, decalcified histological sections were prepared by fixation of the femur’s heads containing the implants in 10% neutral buffered formalin for one week followed by complete bone decalcification in 8% trichloroacitic acid. The decalcified bone segments containing the implants were processed following the routine procedures [30]. Removal of the osseointegrated implants was succeeded through cutting of two opposite longitudinal incisions around each implant. Then, each bone segment was separated into two halves to allow the implant removal. Each half of bone was embedded in a box of molten was to obtain 5-µm longitudinal sections of the parallel edge of bone facing the implant space. The sections were stained with hematoxylin and eosin stain (H&E) for histological examination with light microscope.

### Histomorphometric analysis

After six-week healing period, the undecalcified bone histological sections were prepared as follows: bone blocks containing the implants were subjected to fixation and dehydration, imbedded in transparent methyl methacrylate monomer and finally sectioned in a precision cutting machine using a diamond-coated disc producing 150-μm-thick sections. Sections were polished using silicon carbide and stained using Stevenel’s Blue and van Gieson picrofuchsin. Histomorphometric analysis and calculation of bone-to-implant contact percentage (BIC%) were performed on the mid-section of each implant using digital images obtained from a stereo stereomicroscope (Olympus imaging digital camera, model E.330 DC 7. 4 V, Japan). The images were then analyzed using computer software program (Olypus. Cell ˆA). Mature bone stained red in contact with implant diameter was measured as a percentage of the entire implant diameter to calculate bone implant contact percent of each test group [31].

### Sample size estimation

Sample size was estimated assuming 5% alpha error and 80% study power. Based on results obtained from pilot study, the mean %BIC was 77.4 ± 0.6, 80.1% ± 1.2 and 83.6% ± 2.6 for PEEK implants treated with Nd:YAG laser only, Nd:YAG laser plus UV and Nd:YAG laser plus PRF, respectively. To ensure enough power, sample size was based on difference between Nd:YAG laser plus UV and Nd:YAG laser plus PRF using the highest SD = 2.6. The minimum sample size was calculated to be 10 implants per subgroup, increased to 12 implants to make up for processing errors. The sample was calculated using Gpower3.0.10.

### Statistical analysis

Data were presented as mean, standard deviation (SD), 95% confidence interval (95% CI). Data were explored for normality by using Shapiro–Wilk test. Since data were normally distributed, comparison between the study groups was done using one-way analysis of variance (ANOVA) and followed by Tukey’s test as post hoc after applying Bonferroni correction of multiple comparisons. Two-sided *p* values less than 0.05 were considered statistically significant. Analysis was done using IBM SPSS (Statistical Package for the Social Science; IBM Corp) version 25 for Microsoft Windows.

## Results

**Fig. 1:**
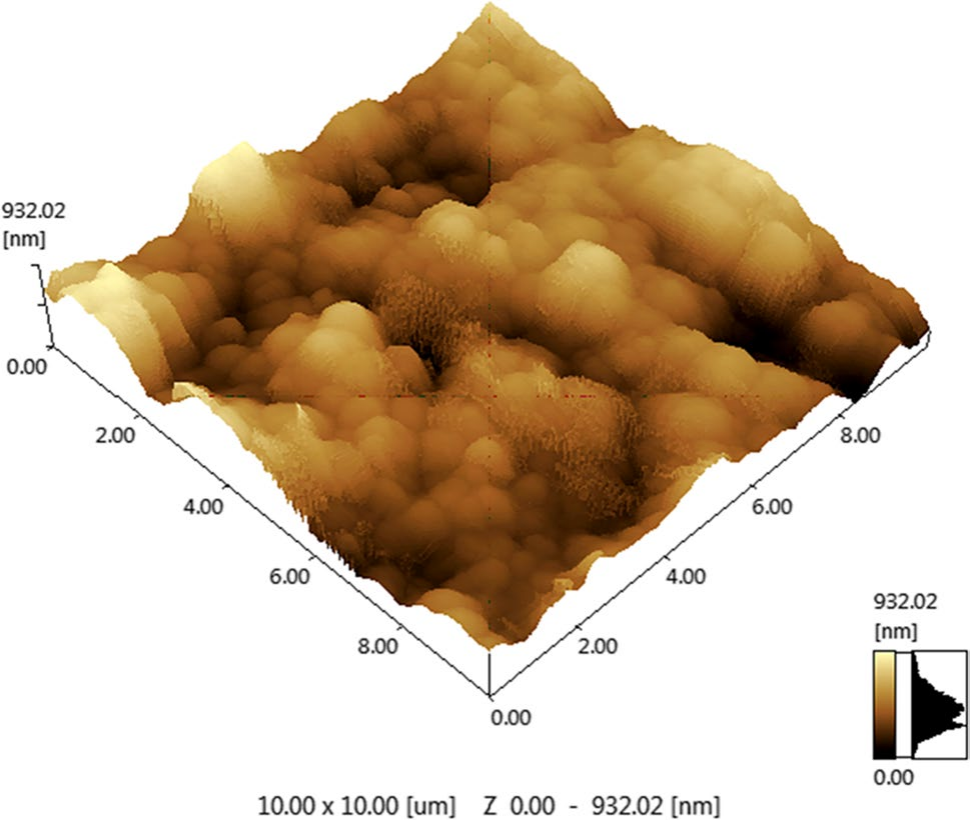
Atomic force microscope image showed the surface roughness nano-topography of the Nd:YAG laser engineered PEEK surfaces

Regarding the surface roughness assessment of Nd:YAG laser nano-topographical engineering for PEEK surfaces, the atomic force microscope displayed the surface roughness parameter (Ra) values in nanometer wherein the mean value of (Ra) was 125.179 nm (SD = 0.41). Additionally, surface roughness nano-topography for each specimen was automatically displayed and represented by colored 3D images (Fig. 1).

**Fig. 2:**
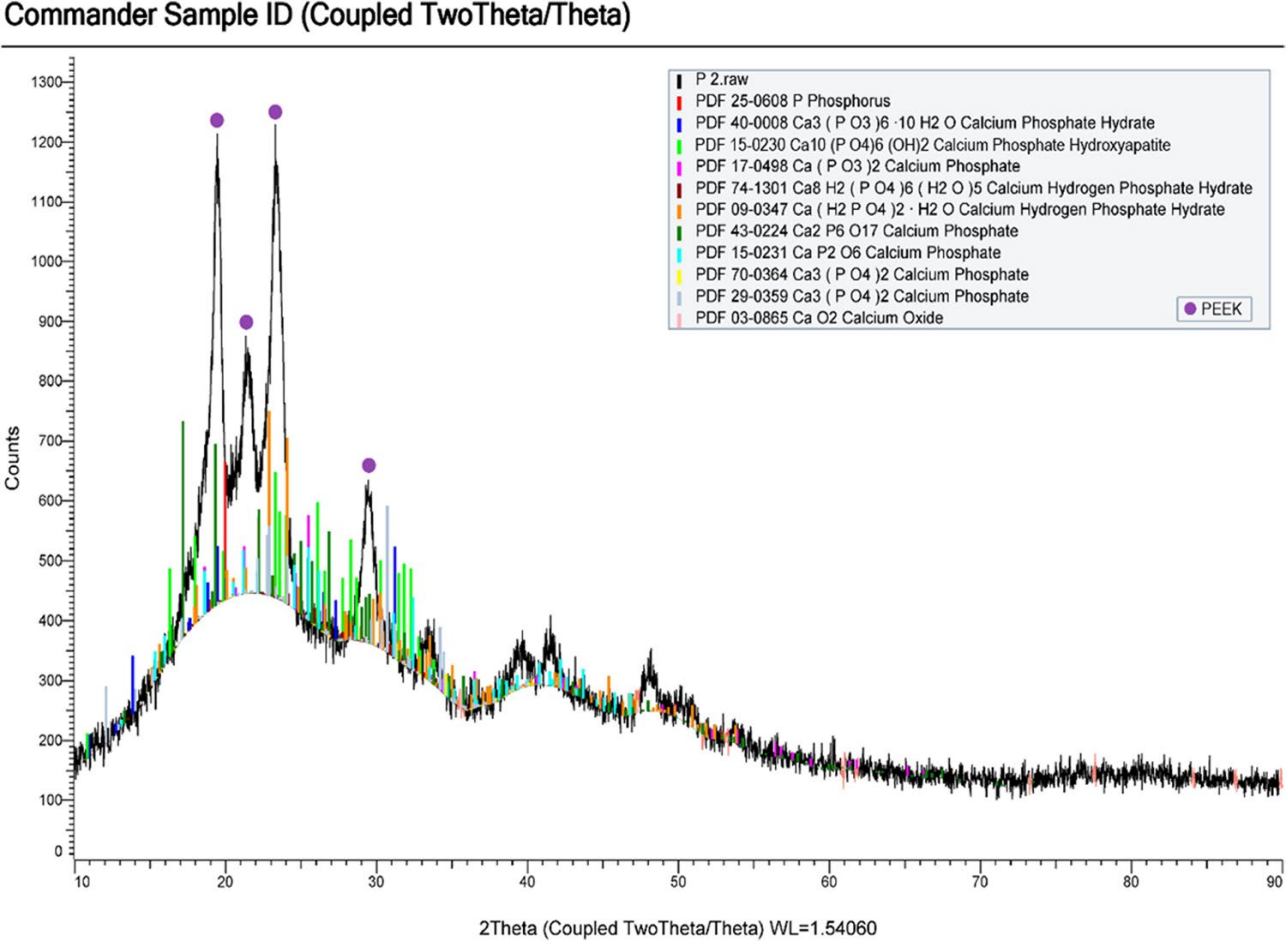
XRD spectrums of Nd:YAG laser nano-topographical engineered PEEK discs in combination with UV or PRF surface modification approaches socked for 4 weeks in simulated body fluid

Concerning the *in vitro* biomimetic–bioactivity test, the X-ray diffraction (XRD) patterns of the Nd:YAG laser nano-topographical engineered PEEK surfaces, either the control surfaces or the Nd:YAG laser engineered PEEK surfaces in combination with UV and PRF, revealed the distinctive X-ray diffraction peaks of PEEK represented as (110), (111), (200) and (211). Also, other diffraction peaks of different calcium phosphate phases were obviously detected included; Ca3 (PO3)6 10H2O – Ca(PO3)2 – Ca2P6O17 – CaP2 O6 – Ca3(PO4)2 and Ca8 H2(PO4)6 (H2O)5. Furthermore, a creditable finding was detected by the evidently distinguished XRD characteristic patterns of hydroxyapatite crystal phase signified as (211), (112) and (300) peaks, Fig. 2.

**Fig. 3:**
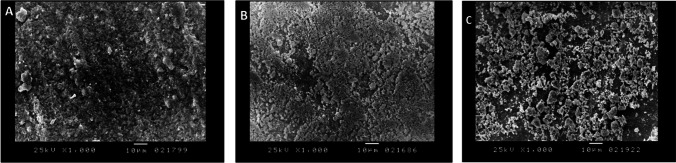
**A**–**C**: SEM images 1000 × demonstrating the 4 weeks in vitro biomimetic–bioactivity assessment of Nd:YAG laser nano-topographical engineered PEEK surfaces. A showed few calcium phosphate crystals scattered over homogenous nano-roughness topography of control laser engineered PEEK surfaces. **B** and **C** demonstrated the apparent accumulations of the apatite minerals all over the nano-topographical modified engineered PEEK surfaces either with UV (**B**) or PRF (**C**). Note: The eminent calcium phosphate aggregations on the PRF modified surfaces

Scanning electron microscope (SEM) images of the control laser engineered PEEK surfaces and those PEEK surfaces modified with UV or PRF schemes showed the aggregations of calcium phosphate minerals found to be dispersed on the engineered PEEK surfaces. A respective difference was observed between the control and both modified PEEK surfaces either with UV or PRF as few calcium phosphate crystals were detected on the control surfaces while apparent masses of apatite minerals significantly covered the modified engineered PEEK surfaces. On the other hand, it could be observed that aggregates of calcium phosphate minerals were more perceptibly spread out on the nano-topographical PEEK surfaces modified with PRF comparing to UV modified PEEK surfaces (Fig. 3A–C).

**Fig. 4:**
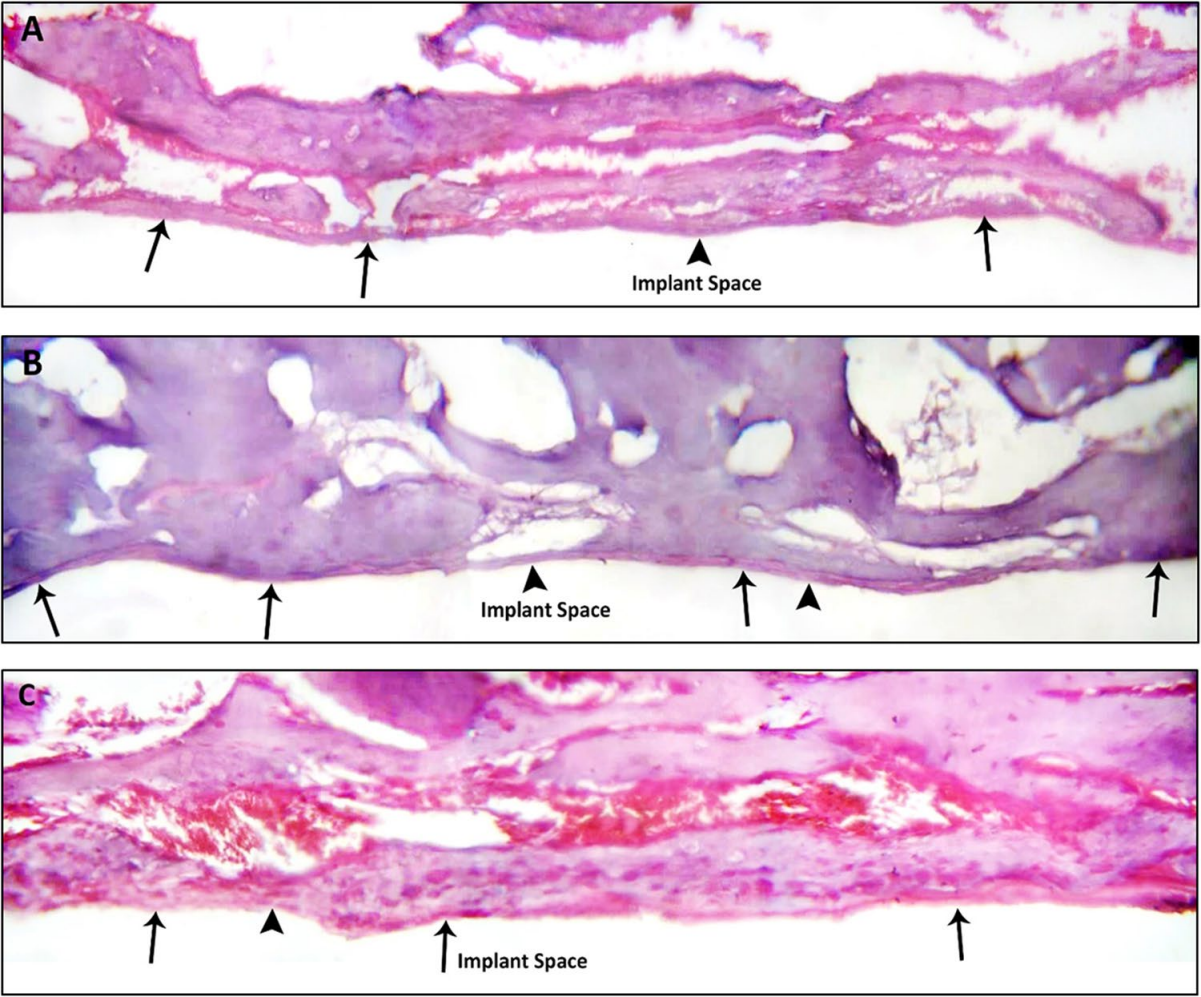
**A**–**C**: Light micrographs (LM), H&E, × 400. The control Nd:YAG laser engineered PEEK implant surfaces showing the segments of contact and the interruption zones (arrows). Note: the interrupted cementing line configuration bordering the interface between the implant space and the bone aspect (arrow head) (**A**). The Nd:YAG laser/UV engineered PEEK implant surfaces display broader and more confluent areas of bone at the implant space (arrows). Note: the bone maturity and the continuous cementing line at the interface (arrow heads) (**B**). The Nd:YAG laser/PRF engineered PEEK implant surfaces show a continuous line of contact between the assumed implant face and the bone. The bone exhibits high organizational quality with a limited area of interruption revealing the continuity of the cementing line (arrow) and the limited area of discontinuity of the bone implant assumed outline (arrow head) (**C**)

Histological findings of control laser nano-topographical engineered PEEK implant surfaces revealed poorer osseointegration features in comparison to the UV and PRF modified laser nano-topographical engineered PEEK implant surfaces. These observations of the control surfaces showed the assumed bone implant interface with segments of contact slightly less than the interruption zones. The bony interface was interrupted at some limited spots all over the full circumference of the implants reflected by interruption of the cementing line bordering the interface between the implant space and the bone aspect, while the other modified UV and PRF nano-topographical engineered PEEK implant surfaces revealed superior osseointegration features represented as a generalized increase in the peri-implant bone density with a continuous line of contact between the assumed implant face. Moreover, the bone with evident bone maturity and persistence of the cement line at the interface was an evident observation (Fig. 4A–C).

**Fig. 5:**
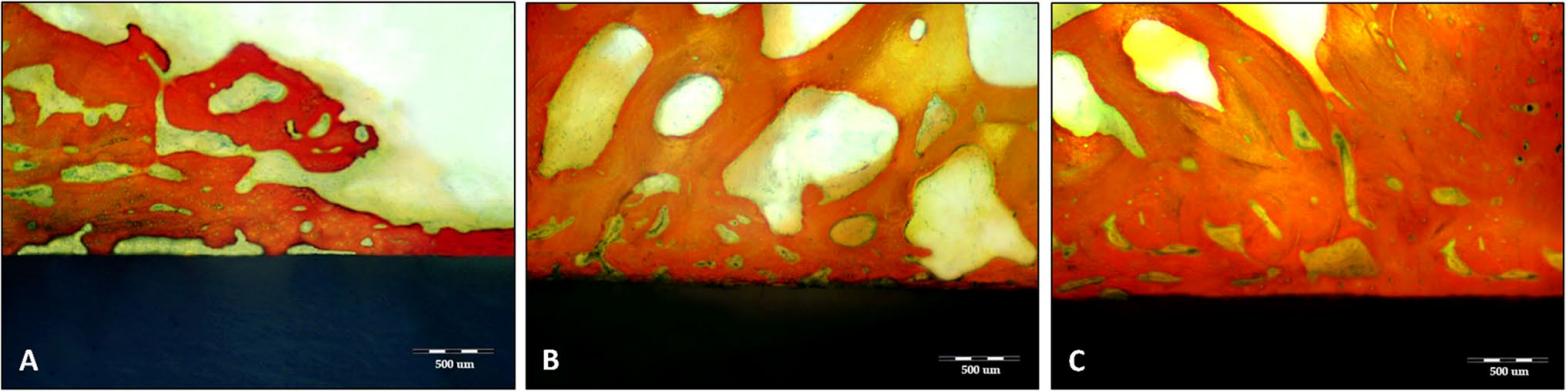
**A**–**C**: Stereomicroscopic images (X: 11 × 10) demonstration the bone implant contact of the Nd:YAG laser nano-topographical engineered PEEK surfaces. Nd:YAG laser engineered surfaces (control group) (**A**), Nd:YAG laser/UV engineered surfaces (**B**) and Nd:YAG laser/PRF engineered surfaces group (**C**). Note: The line of contact between the formed bone and the implant surface for each group reflecting the ascending BIC% from A to C

Histomorphometric outcomes represented by the calculating BIC% for the three studied groups were shown in Table 1. The least BIC% mean value was 56.43 (0.97) recorded for the control Nd:YAG laser engineered PEEK implant surfaces. Then, a significant increase of the BIC% mean value was recorded as 77.30 (0.78) for the Nd:YAG laser/UV engineered PEEK implant surfaces, followed by the highest significant BIC% mean value that was 84.80 (1.29) for the Nd:YAG laser/PRF engineered PEEK implant surfaces. Regarding these outcomes there was a statistically significant increase in the BIC% from the control nano-topographical PEEK implant surfaces to the two modified UV and PRF laser nano-topographical engineered PEEK implant surfaces, as well as there was a statistically significant increase from the modified UV to the modified PRF laser nano-topographical engineered PEEK implant groups (< 0.0001). Stereomicroscopic images of the histomorphometric analysis were captured for screening the BIC% of each studied group as well as the highly organized bony trabecular architectures with the newly formed bone was in direct contact with the implant surface as shown in Fig. 5A–C.

**Table 1:** Mean values of bone-to-implant contact percentage (BIC%) for the study groups

	Nd:YAG laser engineered PEEK surfaces (*n* = 12)	Nd:YAG laser/UV engineered PEEK surfaces (*n* = 12)	Nd:YAG laser/PRF engineered PEEK surfaces (*n* = 12)
Mean (SD)	56.43 (0.97)	77.30 (0.78)	84.80 (1.29)
95% CI	54.88 – 57.96	76.04 – 78.55	82.74 – 86.85
*F* test (*p* value)	801.74 (< 0.0001*)		
Post hoc comparisons	P_1_ < 0.0001*, P_2_ < 0.0001*, P_3_ < 0.0001*		

P_1_: comparison between Nd:YAG laser and Nd:YAG laser + UV, P_2_: comparison between Nd:YAG laser and Nd:YAG laser + PRF, P_3_: comparison between Nd:YAG laser + UV and Nd:YAG laser + PRF

*95% CI* 95% confidence interval

*Statistically significant at value ≤ 0.05

## Discussion

Dental implants have been undergoing a constant flux in terms of ideas and advances to increase implant bioactivity and bone-implant interfacial strength. PEEK has been a prime synthetic competitive candidate among load-bearing dental/orthopedic applications to replace metallic implants [9]. Upon comparison with traditional metal implants, there is less risk of stress shielding caused by material stiffness mismatch between the implant and biological tissue. This is attributed to the similarity of PEEK elastic modulus to that of human bone. Additionally, PEEK is inherently radio-lucent, allowing greater post-operative perceptibility of the surrounding tissue. Its privilege characteristics of heat resistance and chemical compatibility with various sterilization techniques coupled with low-cost molding techniques mark PEEK as a practical and economical material for biomedical device manufacturing [32]. On the other hand, multiple researchers have been investigated to increase the bioactivity of PEEK [33]. Physical or chemical surfaces modification strategies were reported to increase surface energies and encourage binding of biological molecules [34, 35]. Osseointegration is the crucial success goal of implants that depends on topographical and physicochemical properties [36]. Multiple studies have demonstrated that, implant surface properties could be considered the significant key to control the amount and quality of cells adhered to the implant resulting in enhancing implant osseointegration. Consequently, surface chemistry and topography are the most influential properties for cell adhesion. Surface chemistry controlling the implant wettability hence becomes a deciding factor for the protein adsorption and cell adhesion [37]. On the other hand, the implant surface topography and roughness have been heightened during the last four decades to continuously improve the long-term success [14]. Laser modification approach emphasizes on improving the implant osseointegration through generating a nano-scale topographical surface pattern. Accordingly, laser modification could be considered a promising scheme due to their high resolution, high operating speed, low cost and keeping the bulk properties unaltered. Also, laser enhances the implant surface wettability that plays a key role in determining proteins adsorption and cell adhesion on the implant surfaces resulting in improved peri-implant osseointegration [20, 38]. Moreover, it was confirmed by Zheng et al. [39] that the improvement of biocompatibility of the laser textured PEEK surfaces attributed to the formation of polar groups. Another efficacious issue for improving implant osseointegration is the ultraviolet irradiation. It was recognized that ultraviolet irradiation causes removal of implant surface hydrocarbons leading to increased protein binding force, promoting osteoblastic activity and promoting cell attachment and proliferation resulting in enhanced peri-implant bone healing [24]. One of the great challenges is the development of bioactive proteins, which are being used to improve the healing process. Healing is a complex process, which involves cellular organization, chemical signals and the extracellular matrix for tissue repair [40]. Platelet rich fibrin (PRF) consists of an autologous leukocyte-platelet-rich fibrin matrix that could serve as a vehicle in carrying cells involved in tissue regeneration and appears to have a continued release of growth factors in a period between 1 and 4 weeks, hence stimulating the wound healing process in a significant amount of time [26, 41]. Concerning previous prospective, this current study could be considered an innovative trial to improve the bioactivity of PEEK implant surfaces. In this recognition, Nd:YAG laser was the optimum engineering approach to basically adjust the PEEK implant surfaces topography to the nano-scale. Moreover, two different modification techniques either UV or PRF were carefully picked in combination with the nano-topographical engineered PEEK surfaces. Both laboratory and animal testing model assessments were conducted to evaluate the biological responses and osseointegration of bioactive engineered PEEK surfaces. The atomic force microscope was used to determine the surface roughness parameter (Ra) in nanometer resulted in a mean value equal to 125.179 nm (SD = 0.41). This result indicates that application of the Nd:YAG laser leads to successful engineering of the PEEK surfaces topography to the nano-scale. A comparable study conducted a comprehensive survey at three different laser wavelengths: *λ* = 1064, 532 and 355 nm to compare their effect on the roughness and contact angle of PEEK substrates, properties directly related to the cell viability on implants. It was found that the 355-nm laser radiation produced a slight surface melting with the formation of some polar groups [carboxyl (O–C = O) and peroxide (O–O)] on the PEEK surfaces resulting in a potential promotion of cell adhesion onto laser-treated PEEK. A similar effect was observed by exposing PEEK to Q-switched Nd:YAG laser radiation (*λ* = 1064 nm) [42]. The potentiality of this approach to enhance the biological response of biomaterials was mostly studied in metals and mainly titanium alloys [43–45]. Biomimetic–bioactivity test for the present study using SBF is considered a reliable method to evaluate their bone bonding ability [28]. The XRD patterns of the studied groups either the control laser engineered nano-topographical PEEK surfaces or laser engineered PEEK surfaces modified with UV or PRF have shown the distinctive peaks of PEEK substrates, in addition to the significant peaks of some calcium phosphate minerals with the detection of the characteristic peaks of hydroxyapatite. These XRD results confirmed the formation of different apatite minerals including hydroxyapatite on the nano-topographical engineered PEEK surfaces of the three studied groups, and so verifying the in vitro bioactivity of these engineered PEEK surfaces. Besides, SEM images demonstrated accumulations of the apatite minerals on the nano-topographical engineered PEEK surfaces of the three studied groups with a significant difference directly related to the amount of dispersed minerals, where few crystals were observed on the control PEEK surfaces, while many aggregations were noted all over the laser/UV engineered PEEK surfaces with obviously great coverage for the laser/PRF engineered surfaces. Theses surface morphological observations indicated the recognizable bioactivity enhancement of the engineered PEEK surfaces that could be predicted in an ascending pattern from the control group to the laser/UV engineered group to the highest bioactivity for the laser/PRF engineered surfaces. Martin et al. [46] conducted a study to evaluate PEEK used as an implant material after surface modification by electron beam deposition of titanium using SBF to evaluate their bioactivity. The study concluded that PEEK modified by electron beam deposition of titanium had improved bioactivity when compared to unmodified PEEK. Histological findings of the decalcified bone sections demonstrated significant osseointegration features for the three studied groups. Meanwhile, the control nano-topographical engineered PEEK surfaces showed less bonded bone segments at the bone-implant interface in association with an interrupted cementing line configuration. On the other hand, the laser engineered PEEK surfaces in combination with UV or PRF showed more bone bonding areas with a continuous cementing line configuration along the bone-implant interface coupled with prominent bone maturity. Also, it was noted that, the laser engineered PEEK surfaces combined with PRF exhibited superior osseointegration features compared with the laser engineered PEEK surfaces modified with UV. Likewise, histomorphometric outcomes represented by the calculated BIC% for each implant revealed a significant increase in the BIC% mean value among the three studied groups. The least mean value of BIC% was recorded for the control nano-topographical engineered PEEK surface 56.43 (0.97), then significantly increased for the laser/UV engineered PEEK surfaces 77.30 (0.78) to the highest statistically significant mean value for the laser/PRF engineered PEEK surfaces 84.80 (1.29). (< 0.0001). These histological observations and BIC% validated the ability of the nano-topographical engineered PEEK implant surfaces of the three studied groups to interact with the biological bone tissues stimulating the peri-implant bone healing, hence improving their osseointegration. Accordingly, it could be indicated that the bioactivity of PEEK implant surfaces was successfully improved either for the control nano-topographical engineered PEEK implants or laser engineered PEEK implant surfaces in combination with UV and PRF. Furthermore, laser engineered PEEK implant surfaces in combination with PRF could be considered the most bioactive implants comparing to the other two studied groups. It is commendably stated that these histological results were comparable with the SEM observations of apatite minerals formation that were displayed from the in vitro biomimetic–bioactivity test for the studied groups. Guo et al. [47] focused their attention on the roughness effect on the biological activity and osteogenic efficiency of laser-treated surfaces. Femtosecond laser irradiation was used to modify the surface of PEEK implants (with and without the reinforcement of nano-SiO_2_ particles). It concluded that the femtosecond laser surface modification has a significant effect on the PEEK micromorphology and its composite, significantly improving their biological activity. *In vivo* animal study on sheep demonstrated a superior bonding strength of the bone/implant interface during implantation of laser textured treated PEEK implants and concluded enhanced fusion and higher deposition of mineralized matrix observation after 6 months of implantation [48]. Current health-related research is following biomimetic approaches in learning how to engineer new biocompatible materials with nanostructured features [38]. From this prospective, laser nano-topographical engineering approach of PEEK implant surfaces creates a unique bioactive implant surface with nanostructures that mimic the natural environment of cells henceforth able to biologically interact with cells at a molecular level to effectively control the processes of tissue regeneration, such as cell adhesion, proliferation or differentiation and subsequently improved peri-implant bone healing. Although both laser engineered PEEK implants modified with UV or PRF demonstrated improvement of the PEEK implant bioactivity represented by superior osseointegration features and BIC% mean values of 77.30 (0.78) and 84.80 (1.29) respectively, but the laser engineered PEEK implants in combination with UV recorded significantly less BIC% than the laser engineered PEEK implants modified with PRF which recorded the significantly highest BIC%. This could be attributed to the fact that the effect of the UV irradiation could be supplementary to the Nd:YAG laser nano-topographical engineering of PEEK surfaces, because this laser modification approach could be suggested unaccompanied for improving the implant surface wettability [20, 38]. In this sense, modification of the laser engineered PEEK implant surfaces with UV irradiation has been successfully improving bioactivity and osseointegration through the creation of the biomimetic nanostructure implant surfaces in association with modification of the implants surface energy. Similar results of recent studies support the UV irradiation to improve implant surfaces wettability and bioactivity [21–25]. AL Qahtani et al. [37] conducted a study to evaluate the change in the surface wettability of titanium dental implants after UV radiation. The study concluded that UV radiation of different implant surfaces improved their wettability which would lead to improved biological response and improved bone forming capabilities. Regarding the Nd:YAG laser nano-topographical engineered PEEK implant surfaces in combination with PRF, it was reported that the PRF could be considered a source of growth factors involved in osteoblast adhesion, improving subsequent bone healing [27]. From this point, it could be supposed that this enhancement of the implant surfaces bioactivity might be due to a compound modification scheme including Nd:YAG laser resulting in an engineered biomimetic nano-topographical bioactive implant surface in association with alteration of the implant surface energy by increasing the surface wettability, as well as the bioactive protein PRF coat that allows another modification to the implant surfaces by alteration of their chemistry. Therefore, this compound unique scheme for PEEK implant surfaces modification creates an engineered biomimetic/bioactive implant surfaces through topographical, physical and chemical modifications; thereby, the laser/PRF engineered PEEK implant surfaces achieved the superior significant bioactivity and peri-implant bone healing. Different studies discussed the effect of platelet rich plasma (PRP) and platelet rich fibrin (PRF) in dentistry to enhance osseointegration and peri-implant bone healing. Meanwhile, the potential of these studies recognizes titanium and even zirconia implants [26, 29, 31]. AL Qahtani et al. [37] conducted a study to evaluate the change in the surface wettability of dental implants after UV radiation. The study concluded that UV radiation of different implant surfaces improved their wettability which would lead to improved biological response and improved bone forming capabilities. Regarding PEEK implants, there has been a lot of focus on nano-scale coating of PEEK with bioactive apatite and production of bioactive PEEK nanocomposites to enhance their biological responses [49]. To the best of our knowledge, no other studies compared the *in vitro* and *in vivo* bioactivity of PEEK implants following similar design as this present study to improve PEEK implant bioactivity resulting in the production of a simple nano-topographical, biomimetic, bioactive engineered PEEK implant system.

## Conclusions

Within the limitation of this study and based on the outcomes of *in vitro* biomimetic–bioactivity test as well as histomorphometric (BIC%) of *in vivo* animal phase, it might be concluded that Nd:YAG laser could be considered an efficacious system to engineer the PEEK implant surface topography to the nano-scale creating a biomimetic nano-topographical implant surfaces associated with enhanced biological tissue responses confirming the successful modification of the inert surface of PEEK implant to be able to interact with the bone tissues as a bioactive engineered implant system which is directly related to improving osseointegration. Furthermore, it was proved that combination of UV or PRF with this laser nano-topographical engineered bioactive PEEK implant system could be a powerful simple and economic approach to gain more significant improvement of the PEEK implant surface bioactivity accompanying with superior osseointegration, henceforth long-term success.

## Limitations and recommendations

Within the limitation of this research, adding titanium implant as a control group could be recommended for further studies. Likewise, another limitation is that the time of rabbit sacrifice at 6 weeks, hence extended healing periods, may give better figures about implant bioactivity or osseointegration.

### Supplementary Information

Below is the link to the electronic supplementary material.

Supplementary file1 (PDF 540 KB)

## Data Availability

All data presented in the manuscript are available for publication.

## Abbreviations

*PEEK*: Polyetheretherketone
*Nd:YAG*: Neodymium-doped yttrium aluminum garnet
*CAD CAM*: CAD stands for Computer-Aided Design and CAM stands for Computer-Aided Manufacturing
*UV*: Ultraviolet
*PRF*: Platelet rich fibrin
*XRD*: X-ray diffraction analysis
*SEM*: Scanning electron microscopy
*BIC%*: Bone-implant contact percentage
*Ti*: Titanium
*HA*: Hydroxyapatite
*SBF*: Simulated body fluid
*H&E*: Hematoxylin and eosin stain
*PRP*: Platelet rich plasma
