# Supplementary material for: Nano-topographical surface engineering for enhancing bioactivity of PEEK implants (in vitro—histomorphometric study)
Source: Clin Oral Investig. 2023 Oct 17;27(11):6789–99. doi: 10.1007/s00784-023-05291-w (PMC10630241; doi:10.1007/s00784-023-05291-w)
Supplement: Supplementary file 1 — Supplementary file1 (PDF 540 KB) [file 784_2023_5291_MOESM1_ESM.pdf]

جامعة الإسكندرية
ALEXANDRIA UNIVERSITY

ALEXU-IACUC
Institutional Animal Care and Use Committee

Member of ICLAS
http://iclas.org/members/member-list

Review Report

AU	Code of faculty	yy	mm	dd	MS (1)- MD (2) – research (3) - project (4)	Daily serial
	11	19	07	14	3	1

Date of monthly meeting: 14 / 7 / 2019

Name of the researcher: Dawlat Mostafa Ahmed

Name of Department : Dental Biomaterials Department

Type of research: MS ☐ MD ☐ Promotion research √

Title of the research: Nano-Topographical Surface Engineering for Enhancing Bioactivity of PEEK Implants (In vitro – Histomorphometric Study)

Reviewer report:

Accepted according to ethical standards of scientific research √

Accepted after correction ☑ **not accepted** ☐

....
....
....
....
....

Chairperson of AU-IACUC **Chairperson of local IACUC**

Prof Maha Ghanem **Prof.Dr. Maha Abd Elaziz Abou Khadr**

Alexu-IACUC Institutional Animal Care and Use Committee ALEXANDRIA UNIVERSITY

Address: Faculty of Medicine Alexandria University, 17 Champollion Street, El Messalah, Alexandria, Egypt.
Web site:
Email: **Email:**
Email:
